# Morphological analysis of three-dimensional MR images of patellofemoral joints in asymptomatic subjects

**DOI:** 10.1038/s41598-023-42404-7

**Published:** 2023-10-05

**Authors:** Hisako Katano, Nobutake Ozeki, Mitsuru Mizuno, Kentaro Endo, Noriya Okanouchi, Jo Fujita, Jun Masumoto, Hideyuki Koga, Ichiro Sekiya

**Affiliations:** 1 Center for Stem Cell and Regenerative Medicine, Tokyo Medical and Dental University (TMDU), 1-5-45 Yushima, Bunkyo-ku, Tokyo 113-8510 Japan; 2 Kanagawa Institute of Industrial Science and Technology, 3-2-1 Sakado, Takatsu-Ku, Kawasaki, Kanagawa Japan; 3 Fujifilm Corporation, 26-30, Nishiazabu 2-Chome, Minato-ku, Tokyo Japan; 4 Department of Joint Surgery and Sports Medicine, Graduate School of Medical and Dental Sciences, Tokyo Medical and Dental University (TMDU), 1-5-45 Yushima, Bunkyo-ku, Tokyo Japan

**Keywords:** Anatomy, Diseases, Medical research

## Abstract

The existing methods for analyzing patellofemoral (PF) osteoarthritis (OA) are limited. Our purpose was to clarify the frequency, localization, and morphological progression of PFOA by observing three-dimensional (3D) magnetic resonance (MR) images from a cohort population. The subjects were 561 patients aged 30–79 years from the Kanagawa Knee Study who had not visited a hospital for more than three consecutive months for knee symptoms. MR images of the PF joints, separated into the medial and lateral types, were presented in order of the highest to lowest patella cartilage area ratios. Cartilage defects in the patella were detected in 37 subjects (6.6%). Medial lesions (4.6%) were significantly more frequent than lateral lesions (2.0%) (*p* < 0.01). For both medial and lateral lesions, the patellar cartilage defects were divided into confined and unconfined types. The 3D MR images of the PF joint showed that the patellar cartilage defect occurred along each ridge of the femoral trochlea. The 3D MR images revealed a 6.6% prevalence of patellar cartilage defects, higher in the medial than lateral regions. The 3D MR images can easily determine PF morphology and cartilage defect location, making them useful in understanding the pathophysiology and etiology of PFOA.

## Introduction

Patellofemoral (PF) osteoarthritis (OA), like medial femorotibial (FT) OA and lateral FTOA, is a subset of knee OA^1^. The prevalence of PFOA has been studied anatomically^2,3^, radiographically, and by magnetic resonance imaging (MRI)^4^. Anatomical studies of cadavers have revealed detailed changes in the articular surfaces, but an accurate description of the anatomical relationship between the patella and the femoral trochlea is difficult to obtain due to the limited number of cadavers available to observe. Radiographic observation can allow the analysis of large numbers of knees in less time than MRI, but it cannot detect cartilage lesions that occur in the early phase of PFOA. Small lesions in cartilage and bone marrow are detectible in two-dimensional (2D) magnetic resonance (MR) images, but those images cannot provide details of the cartilage lesions as areas. Overall, the existing methods for analyzing PFOA are limited.

One technique that has the potential to resolve these limitations is 3D MR image analysis. Recent advances in analysis systems have made possible the automatic extraction of bone and cartilage areas from knee MR images and the demonstration of three-dimensional (3D) MR images. Using this method, we previously reported a positional relationship between the medial tibial cartilage defect and the medial meniscus by organizing the cartilage defects shown in the 3D MR images in order, from smallest to largest^5^. The cohort data used in that analysis was The Kanagawa Knee Study.

The Kanagawa Knee Study is our epidemiological survey of the knee and includes MRI data for 3D analysis collected from 561 asymptomatic subjects aged 30–79 years. The subjects are current and former desk workers with no history of hospital visits for lower extremity symptoms for more than 3 months. This cohort population can be characterized as an asymptomatic population with a relatively uniform age distribution and gender^6^.

We considered that the availability of 3D MRI data for this cohort could enhance the understanding of the pathogenesis of PFOA by elucidating the onset and progression of asymptomatic PFOA. The purpose of this study was to clarify the frequency of total PFOA, the frequency of lateral and medial PFOA types, and the morphological progression of PFOA. We achieved this by observing the cartilage defects, from the smallest to the largest, detected in 3D MR images in this cohort.

## Methods

### The Kanagawa Knee Study

This study was approved by the Medical Research Ethics Committee of Tokyo Medical and Dental University, and written informed consent was obtained from all subjects. The purpose of the Kanagawa Knee Study is to clarify the epidemiology and natural history of knee OA^6^. The main inclusion criteria are (1) employees of the Kanagawa Prefectural Office, retired employees of the Kanagawa Prefectural Office, or those who work in Kanagawa Prefecture or live in the Tokyo metropolitan area, and (2) those who work at a desk for at least 4 h per day or perform similar work during their employment. The main exclusion criteria are those who have (1) a history of surgery on either the left or right knee and (2) a past history of consecutive visits to the hospital for more than 3 months for symptoms in either the left or right lower extremity. The main data collected for the study include (1) a questionnaire that covers height, weight, history of knee pain, activity level, Knee Injury and Osteoarthritis Outcome Score (KOOS), and Numerical Rating Scale (NRS); (2) MRI and radiographs of the right knee; and (3) urine output^6^.

The present study included 561 asymptomatic volunteers (277 females and 284 males; range 30–79 years, with approximately 50 females and males in each decennial age group) (Supplementary Table 1).

### MRI scanning

The MRI system (Achieva 3.0TX, Philips, Amsterdam, Netherlands) was used at 3.0 T with 16-channel coils. The sagittal plane of the knee joint was acquired to obtain both a fat-suppressed spoiled gradient echo sequence (SPGR) image and a proton density–weighted (PDW) image, with total scan durations of 7 min 17 s and 7 min 30 s, respectively. For both images, sagittal images were obtained at an in-plane resolution of 0.3 × 0.3 mm, a partition thickness of 0.3 mm (320 slices), and a field of view (head to tail × anterior to posterior) of 150 × 150 mm^7,8^.

### 3D MR images

The software used for the MRI analyses was a 3D image analysis system volume analyzer (SYNAPSE VINCENT 3D, Collaborative version, FUJIFILM Corporation). A 3D convolutional neural network (3D-CNN) algorithm for segmentation of bone, cartilage, and the region of interest (ROI) was constructed based on U-Net containing an encoder and a decoder. The outputs were probability maps of the target regions, including the background region. For each pixel in the image, a number was assigned as the region label for that pixel, and the segmentation of the image was obtained^6^.

**Figure 1:**
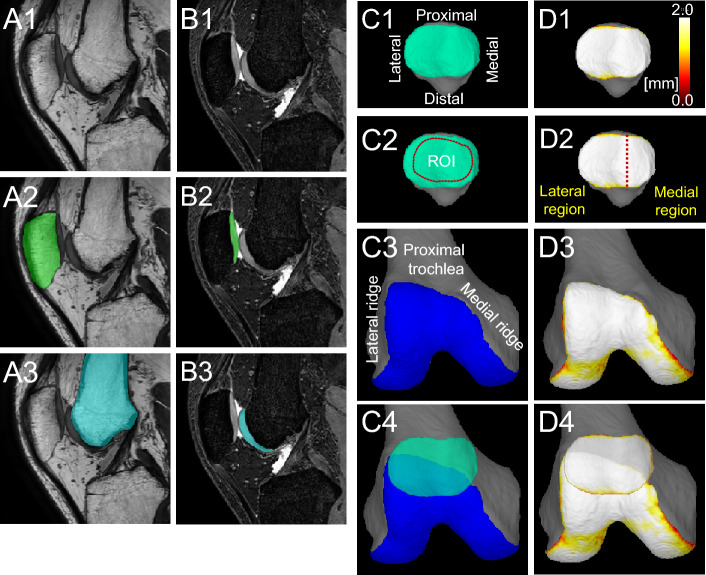
3D MRI analysis of the patellofemoral joint of a representative control knee on the right side. (**A**) Proton density–weighted (PDW) images of the sagittal plane. From the plain images (1), the bone regions were automatically segmented and colored green for the patella (2) and light blue for the femur (3). (**B**) Fat-suppressed spoiled gradient echo sequence (SPGR) images of the sagittal plane. From the plain images (1), the cartilage regions were automatically segmented and colored green for the patella (2) and light blue for the femur (3). (**C**) 3D images of the PF cartilage. The patellar cartilage surface of the right knee is shown inverted from left to right (1). Inner margins of 2 mm of the original or predicted original patellar cartilage are set as the ROI (2). The long axis of the femur is tilted 45°, and the anterior surface of the femoral trochlear cartilage is shown in blue (3). The patellar cartilage is superimposed on the femoral trochlear cartilage (4). (**D**) Cartilage thickness mapping of the PF joint. Patellar cartilage thickness is shown in color (1). The medial 2/5 of the patellar cartilage is defined as the medial region, and the lateral 3/5 as the lateral region (2). The femoral trochlear cartilage thickness is shown in color (3). The patellar cartilage thickness map is superimposed on the femoral trochlear cartilage thickness map (4).

The PDW images were used for automatic segmentation of the bone region (Fig. 1A) and the SPGR images were used for automatic segmentation of the cartilage region (Fig. 1B). The 3D image was then reconstructed (Fig. 1C). The patellar cartilage was rotated and tilted to maximize the patellar cartilage area and projected onto a plane. The femoral cartilage was rotated so that the posterior condylar axis was horizontal and tilted, and the long axis of the femur was tilted 45° and then projected onto a plane. The patellar cartilage was shown inverted medially and laterally in the 3D image so that the patellar cartilage could be overlapped onto the femoral cartilage. The software provided cartilage thickness mapping by displaying cartilage thickness as a color scale, with thick areas represented in white and thin areas in red (Fig. 1D). The thickness of the cartilage was defined as the unit volume of the cartilage and included a vertical line drawn across the subchondral surface divided by the unit area of the subchondral surface.

The 3D MR images of the patellar cartilage were examined for all subjects, and those with cartilage defects were again observed at those points in the 2D MR images (Supplementary Fig. 1).

The cartilage in the femoral trochlea was evaluated only in knees with cartilage defects in the patella. The femoral cartilage was projected in two dimensions under the conditions described above, and the presence or absence of cartilage defects was subjectively evaluated based on the cartilage thickness mapping images. Defect locations were largely categorized into defects that continued to the edge of the trochlea cartilage and defects that did not.

### Medial and lateral types of patellar cartilage defects

A cartilage defect area with the center located in the medial 2/5 of the patellar cartilage area was classified as a medial type, while one located in the lateral 3/5 of the patellar cartilage defect area was classified as a lateral type (Fig. 1D2).

### Confined and unconfined cartilage defects

A patellar cartilage defect that did not extend to the boundary of the original cartilage was classified as a confined defect, while a defect that extended to the boundary of the original cartilage was classified as an unconfined defect.

### Cartilage area ratio in the patella

The software automatically set the ROI of the patellar cartilage to 2 mm inside the cartilage (Fig. 1C2). In cases where the outer margin of the patellar cartilage was deficient, the ROI was manually adjusted based on the bone morphology. The software also automatically computed a “cartilage area ratio,” which represented the ratio of the cartilage area to the total area of the ROI. A cartilage area ratio value of 1 indicates that cartilage covers the entire ROI, whereas a cartilage area ratio value of 0 indicates that no cartilage covers the ROI.

MR images of all subjects were displayed with cartilage area ratios and presented in order of the highest to lowest values. They were further separated into medial and lateral types, as well as confined and unconfined cartilage defects.

### Statistical analysis

The difference in the population proportions between medial lesions and lateral lesions in the patellar cartilage was examined by “hypothesis testing for the difference in the population proportions” using the BellCurve software for Excel (Social Survey Research Information Co., Ltd. Tokyo, Japan). A value of *p* < 0.05 was considered statistically significant.

### Statistics and biometry

The difference in the population proportions with medial lesions versus lateral lesions in the patellar cartilage was examined by “hypothesis testing for the difference in the population proportions” using the BellCurve software for Excel (Social Survey Research Information Co., Ltd. Tokyo, Japan). Since this study did not use any complicated statistical methods, we did not include a statistician as a co-author.

### Methodology

1. Retrospective, 2. prognostic study, 3.performed at one institution.

### Ethical approval

This study was approved by the Medical Research Ethics Committee of Tokyo Medical and Dental University. All methods were performed in accordance with the relevant guidelines and regulations. The protocols were enrolled in a database of the National University Hospital Council of Japan (UMIN000032826) on 01/06/2018 and disclosed. The first subject (Registration Number: KS-001) was included in the study 28/09/2018. The last subject (Registration Number: KS-573) was included on 05/09/2019.

### Informed consent

Written informed consent was obtained from all participants.

## Results

### Incidence of patella cartilage defects from 3D MRI

**Figure 2:**
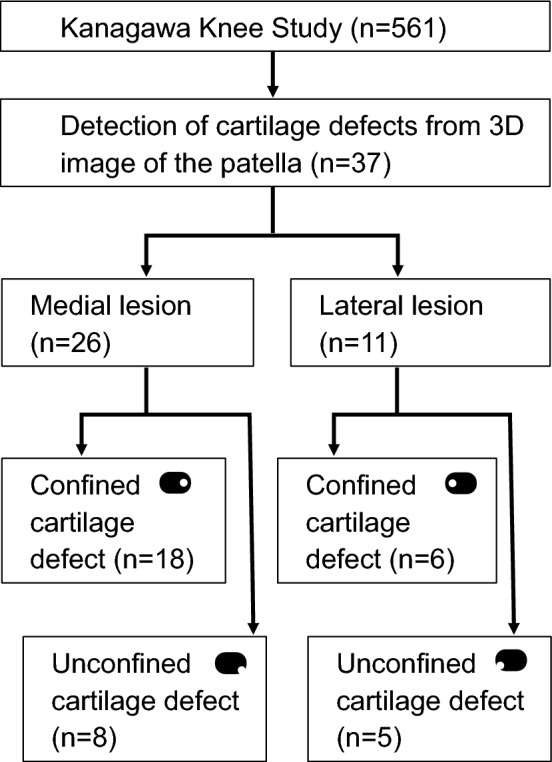
Number of patella cartilage defects from 3D MRI. The Kanagawa Knee Study consisted of 561 subjects. Cartilage defects were detected on 3D images of the patella in 37 subjects. Patellar cartilage defects were located in the medial region in 26 subjects and classified as confined cartilage defects in 18 subjects and unconfined cartilage defects in 8 subjects. Patellar cartilage defects were located in the lateral region in 11 subjects and classified as confined defects in 6 subjects and unconfined cartilage defects in 5 subjects.

Of the 561 subjects (277 females and 284 males), cartilage defects were detected on 3D images of the patella in 37 subjects (6.6%), consisting of 22 females and 15 males (Fig. 2). In terms of gender age groups, 4 subjects (11%, 2 females, 2 males) were in their 50 s, 12 (32%, 8 females, 4 males) were in their 60 s, and 21 (57%, 12 females, 9 males) were in their 70 s, and none were in the 30–49 age group for either sex (Supplementary Table 2). All cartilage defects in the 3D MR images could be confirmed in the 2D MR images (Supplementary Figs. 2, 3).

A medial lesion was detected in 26 subjects (4.6%) and a lateral lesion in 11 subjects (2.0%), showing that medial lesions were significantly more frequent (*p* < 0.01). The medial lesions included confined cartilage defects in 18 subjects (69%) and unconfined cartilage defects in 8 subjects (31%). The lateral lesions included confined cartilage defects in 6 subjects (55%) and unconfined cartilage defects in 5 subjects (45%) (Fig. 2).

### Medial patellar cartilage lesions

**Figure 3:**
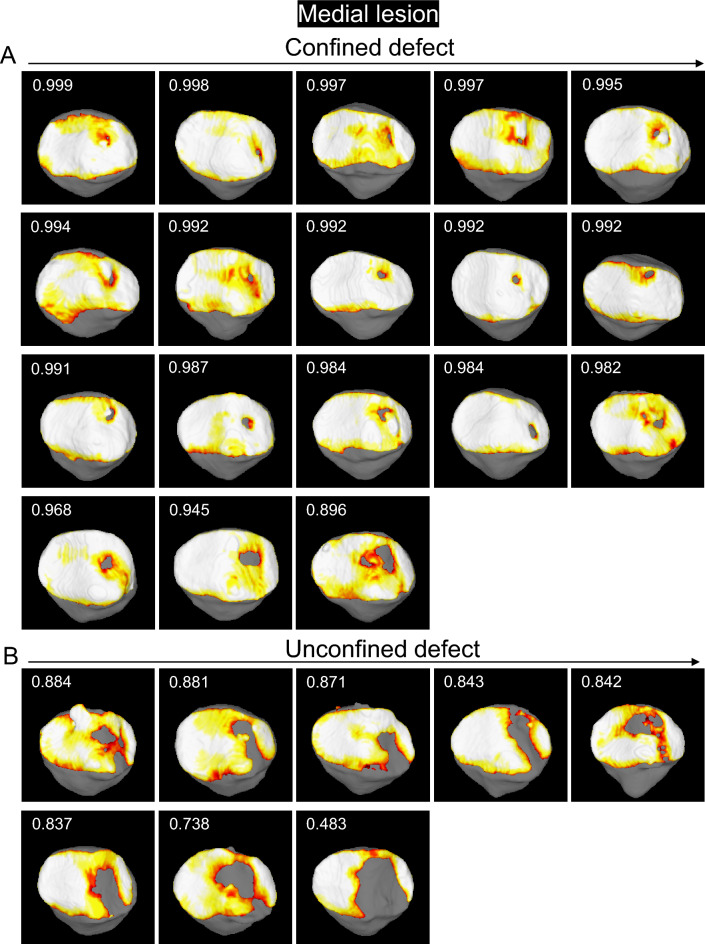
Cartilage thickness mapping of the patella with a cartilage defect in the medial region. (**A**) Confined type where the defect does not extend to the original boundary of the cartilage. The values in the upper corner indicate the cartilage area ratio, and each image is placed in order from highest to lowest. (**B**) Unconfined type, where the defect extends to the original boundary of the cartilage.

The patellar cartilage defect in the confined type was located in the center of the medial facet and the surrounding area, according to the observations made by following the decreasing order of cartilage area ratios in the patella (Fig. 3). The unconfined defect type was further connected distally and medially.

**Figure 4:**
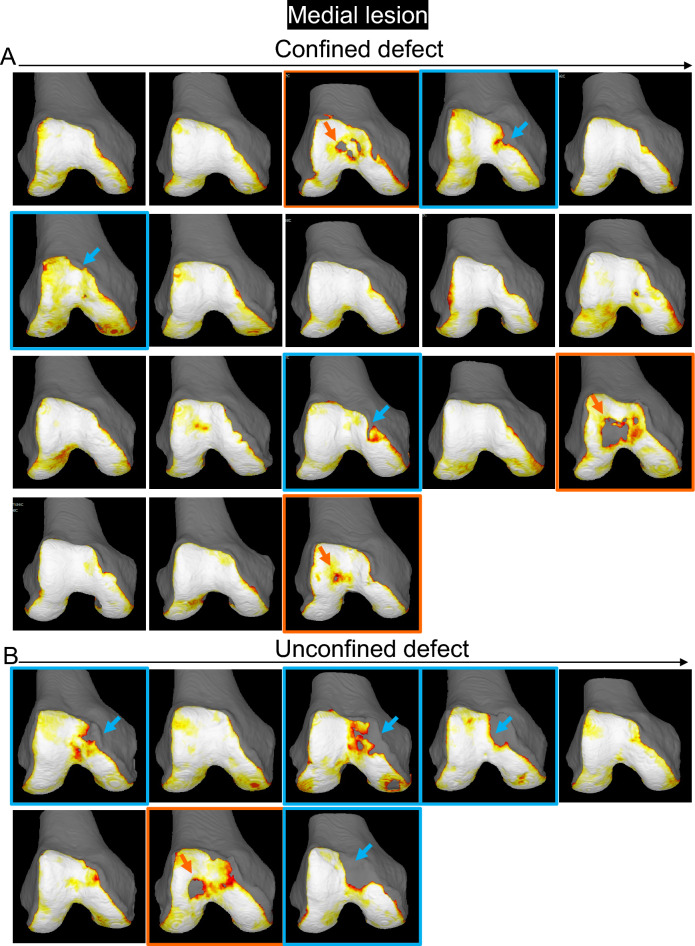
Cartilage thickness mapping of the femoral trochlea in the knee with a patella cartilage defect in the medial region. (**A**) Confined type, where the defect of the patellar cartilage does not extend to the original boundary of the cartilage. (**B**) Unconfined type, where the defect of the patella extends to the original boundary of the cartilage. The femoral cartilage defect along the proximal or medial edge is indicated by a blue arrow, and the panels are surrounded in blue. The femoral cartilage defect at the center of the femoral trochlear is indicated by an orange arrow, and the panels were surrounded in orange.

A femoral cartilage defect along the medial edge was observed in 3 of 18 (17%) of the confined type and 4 of 8 (50%) of the unconfined type (Fig. 4, Supplementary Fig. 2). Conversely, a femoral cartilage defect at the center of the femoral trochlear cartilage was observed in 3 of 18 (17%) of the confined type and 1 of 8 (13%) of the unconfined type.

**Figure 5:**
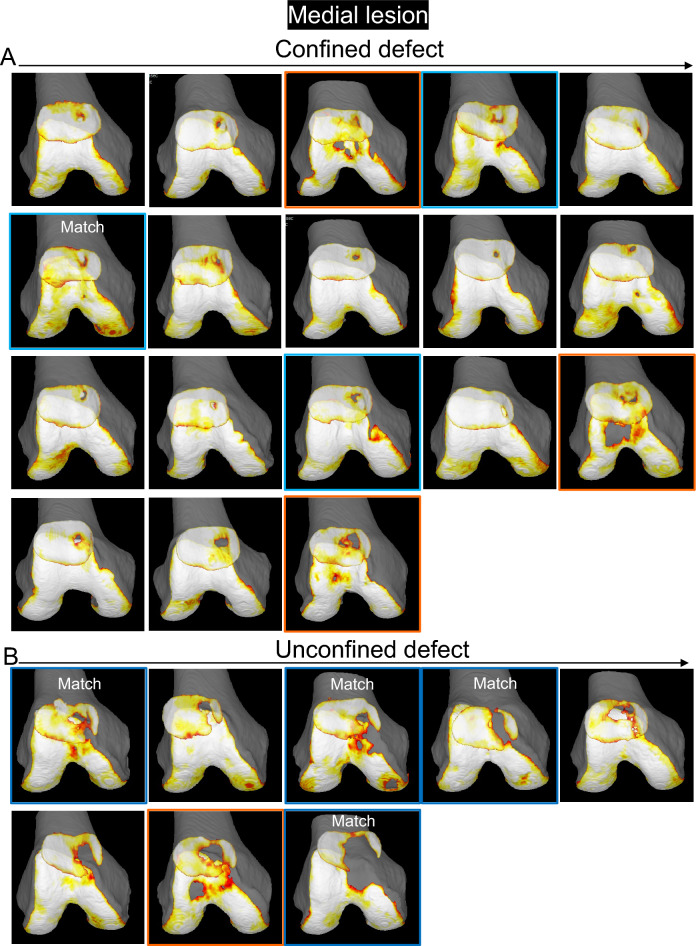
Cartilage thickness mapping of the PF joint with a patella cartilage defect in the medial region. (**A**) Confined type, where the defect of the patellar cartilage does not extend to the original boundary of the cartilage. Each image is placed in the same order as in Fig. 4. Images framed in blue show femoral cartilage defects along the proximal or medial edge, and those framed in orange show femoral cartilage defects at the center of the femoral trochlear cartilage. (**B**) Unconfined type, where the defect of the patella extends to the original boundary of the cartilage.

The 3D MR images of the PF joint showed that the patellar cartilage defect was located around the proximal end of the medial edge of the trochlear cartilage in the confined type and along the medial edge of the trochlear cartilage in the unconfined type (Fig. 5). In the confined type, the patellar cartilage defect matched the femoral cartilage defect in only 1 of 6 cases (17%) that had any defects in the femoral cartilage. Conversely, in the unconfined type, the patellar cartilage defect matched the femoral cartilage defect in 4 of 5 cases (80%) that had any defects in the femoral cartilage.

### Lateral patellar cartilage lesions

**Figure 6:**
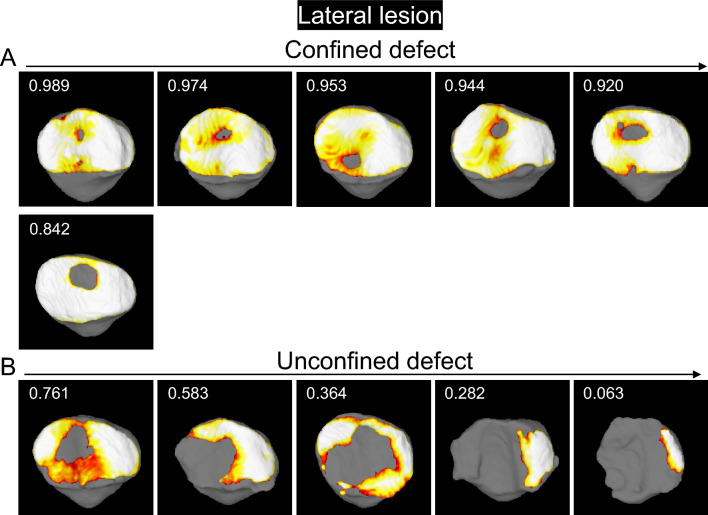
Cartilage thickness mapping of the patella with a cartilage defect in the lateral region. (**A**) Confined type, where the defect does not extend to the original boundary of the cartilage. The values in the upper left corner indicate the cartilage area ratio, and each image is placed in order from highest to lowest. (**B**) Unconfined type, where the defect extends to the original boundary of the cartilage.

The patellar cartilage defect in the confined type was located in the center of the lateral facet and the surrounding area according to the observations made by following the order of the cartilage area ratio in the patella (Fig. 6). The defect was further connected distally and laterally in the unconfined type.

**Figure 7:**
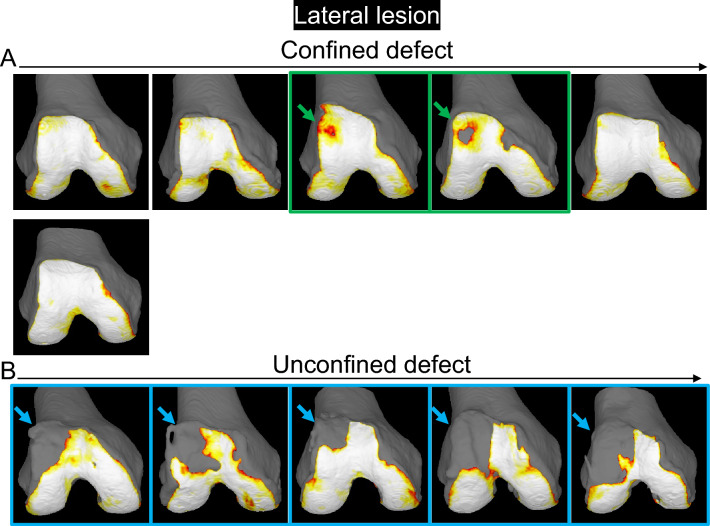
Cartilage thickness mapping of the femoral trochlea in the knee with a patella cartilage defect in the lateral region. (**A**) Confined type, where the defect of the patellar cartilage does not extend to the original boundary of the cartilage. (**B**) Unconfined type, where the defect of the patella extends to the original boundary of the cartilage. The femoral cartilage defect that does not reach the edge of the trochlea is indicated by a green arrow, and the panels are surrounded in green. The femoral cartilage defect that reaches the edge of the trochlea is indicated by a blue arrow, and the panels are surrounded in blue.

A femoral cartilage defect was observed at the proximal lateral area that did not reach the edge of the trochlea in 2 of 6 (33%) of the confined type (Fig. 7, Supplementary Fig. 3). By contrast, a large defect at the femoral cartilage was observed at the proximal lateral area that reached the edge of the trochlea in all 5 of 5 (100%) of the unconfined type.

**Figure 8:**
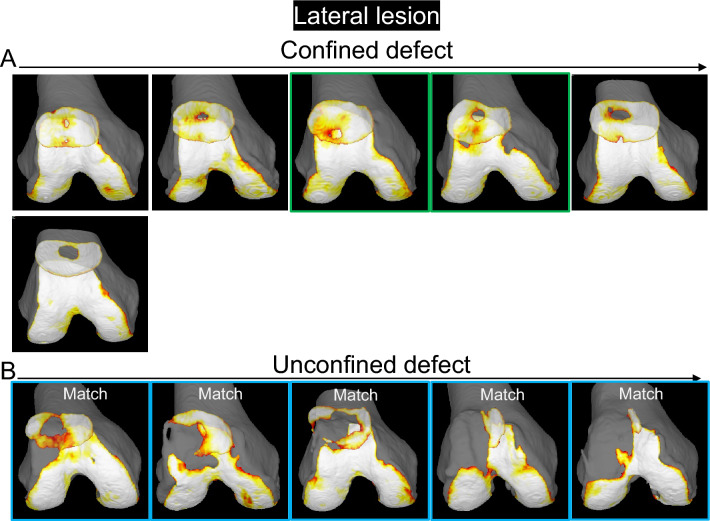
Cartilage thickness mapping of the PF joint with a patella cartilage defect in the lateral region. (**A**) Confined type, where the defect of the patellar cartilage does not extend to the original boundary of the cartilage. Each image is placed in the same order as in Fig. 7. Images framed in green show that the femoral cartilage defect does not reach the edge of the trochlea, and those framed in blue show that the femoral cartilage defect reaches the edge of the trochlea.

The 3D MR images of the PF joint showed that the patellar cartilage defect was located at the proximal end of the trochlear cartilage in 5 of 6 (83%) in the confined type and along the proximal and lateral edge of the trochlear cartilage in the unconfined type (Fig. 8). In the confined type, the patellar cartilage defect matched the femoral cartilage defect in 0 of 2 cases (0%) that had any defects in the femoral cartilage. By contrast, the patellar cartilage defect matched the femoral cartilage defect in 5 of 5 cases (100%) that had any defects in the femoral cartilage.

The 2D MR images indicated hypoplasia of the patellar groove, a lateral shift of the patella, or a lateral tilt of the patella in all subjects (Supplementary Fig. 2).

## Discussion

The frequency of patellar cartilage defects was 6.6% in our 3D MRI analysis of 561 asymptomatic subjects. This is the first report to reveal the frequency of patellar cartilage defects in 3D MR images.

A review article by Hart et al.^4^ reported a frequency of PFOA, based on radiography, of 38% (28–50%) (mean proportion; 95% CI) in nine studies of community-based populations and 17% (6–33%) in four studies of healthy individuals, where the definition of healthy subjects was no history of surgery, meniscus, or ligament damage^9^, no knee pain or injury^10^, no knee symptoms on physical examination^11^, and women in general as controls for female ex-athletes^12^. Based on MRI data, the frequency of any cartilage defects defined as cartilage abnormality, cartilage defect, full cartilage thickness loss, cartilage pathology, or cartilage lesion was 44% (25–65) in three studies of community-based populations, and the frequency of any cartilage defects was 40% (19–63) in two studies of healthy individuals^4^. Healthy subjects were defined as Osteoarthritis Initiative (OAI) participants with bilateral Kellgren/Lawrence (K/L) scores of 0^13^ and participants who had not undergone arthroscopic partial meniscectomy^14^. Our numbers are lower than those reported previously, probably because our definition of a cartilage defect is full cartilage thickness loss in 3D MR images, and because the subjects were current and former desk workers with no history of hospital visits for lower extremity symptoms for more than 3 months.

A medial lesion was detected in 26 subjects (4.6%) and a lateral lesion in 11 subjects (2.0%), showing that medial lesions were significantly more frequent (*p* < 0.01). Among the previous reports that used community-based radiographic analysis, three reports showed that lateral lesions were more frequent than medial lesions^15–17^, but no reports showed the opposite results. Semiquantitative MRI analyses using WORMS and MOAKS in community-based populations revealed three reports that showed more frequent occurrences of medial lesions than lateral lesions^18–20^, but no opposite findings. Anatomical studies of cadaveric knees included two reports that showed a greater frequency of medial lesions than lateral lesions^2,3^, but no opposite results. MRI and anatomical observations are more accurate than radiographic assessments for evaluating cartilage lesions; consequently, the patellar cartilage will be more susceptible to OA on the medial side than on the lateral side.

For a medial lesion of the patella, sequential 3D MR images in decreasing order of the cartilage area ratio demonstrated that the patellar cartilage defect was located in the center of the medial facet in knees with a higher cartilage area ratio, multiple defects were located in and around the center of the medial facet in knees with a lower cartilage area ratio, and defects were found in and around the medial facet or as multiple defects in the following pathway in the unconstrained type with a lower cartilage area ratio. The 3D MR images of the femoral cartilage with the patellar cartilage showed that the cartilage defect of the patella was located around the proximal end of the medial edge of the trochlear cartilage in the confined type and along the medial edge of the trochlear cartilage in the unconfined type. This suggests that the patellar cartilage defect occurred around the proximal end of the medial edge of the trochlear cartilage and along the medial edge of the trochlear cartilage in the unconstrained type. Patellar cartilage defects corresponded to femoral cartilage defects in 1 of 6 (17%) of the confined type and 4 of 5 (80%) of the unconfined type. This suggests that mirror lesions occur at around 20° of knee flexion during MRI scanning. Conversely, in 5 of 6 (83%) of the confined type and 1 of 5 (20%) of the unconfined type, the patellar and femoral cartilage defects did not match, and the femoral cartilage defect was located more distally. This suggests that the patella shifts medially in the more flexed position of the knee, leading to a mirror lesion. The finding of a femoral cartilage defect located in the center of the trochlea suggests that the patella does not shift medially, as it does in the other cases during flexion, and this leads to a mirror lesion.

For the lateral lesion of the patella, examination of sequential 3D MR images in decreasing order of the cartilage area ratio demonstrated that the patellar cartilage defect was located in the center of the lateral facet and the surrounding area in the confined type, whereas it expanded further distally and laterally in the unconfined type. This suggests that the patellar cartilage defect initiates at the center of the lateral facet and that if environmental factors lower the cartilage area ratio, the defect may extend to the center and then further distally and laterally. The 3D MR images of the femoral cartilage with the patellar cartilage showed that the cartilage defect of the patella was located around the proximal end of the trochlear cartilage in the confined type and along the proximal and lateral edges of the trochlear cartilage in the unconfined type. This suggests that when an unconstrained defect occurs, the patellar cartilage defect occurred around the proximal end of the trochlear cartilage and along the lateral edge of the trochlear cartilage.

We analyzed cartilage defects of the patella by dividing them into medial and lateral lesion types. We did this because we thought that the pathophysiology differed between the two types. A patella that shifts medially during knee motion would be more likely to have a medial defect, while a patella that shifts laterally would be more likely to have a lateral defect. This study suggests that the medial lesion type would be caused by stress on the medial edge of the trochlea and that the lateral lesion type would be caused by mechanical stress imposed on the lateral edge of the trochlea.

Our study had two limitations. One was that the cartilage defect area with a center located in the medial 2/5 of the patellar cartilage area was classified as the medial type, whereas one with a center in the lateral 3/5 of the patellar cartilage defect area was classified as the lateral type. This arbitrary boundary does not correspond to the vertical center ridge of the patella. In addition, in our classification, defects in the vertical center ridge of the patella are classified as a lateral type. Nevertheless, lateral lesions were fewer in number than medial lesions. A second limitation was that we did not mention medial or lateral displacement of the patella or elucidate its cause. A displacement involves malalignment of the PF joints or patellar instability. A comparison of these factors between groups with and without cartilage defects in this population will be the topic of our next study.

## Conclusion

The 3D MR images revealed a frequency of patella cartilage defects of 7% in 561 asymptomatic subjects. Medial patella lesions occurred more frequently than lateral lesions. 3DMRI, which can easily determine patellofemoral morphology and cartilage defect location, is useful in understanding the pathophysiology and etiology of PFOA.

### Supplementary Information

Supplementary Figures.

Supplementary Tables.

Supplementary Legends.

## Data Availability

The datasets used and/or analyzed during the current study are available from the corresponding author on reasonable request.

## Abbreviations

PF: Patellofemoral
OA: Osteoarthritis
MR: Magnetic resonance
MRI: Magnetic resonance imaging
3D: Three-dimensional
2D: Two-dimensional
ROI: Region of interest
DICOM: Digital imaging and communications in medicine
WORMS: Whole-organ magnetic resonance imaging score
MOAKS: MRI osteoarthritis knee score
