# Supplementary material for: Morphological analysis of three-dimensional MR images of patellofemoral joints in asymptomatic subjects
Source: Sci Rep. 2023 Oct 5;13:16750. doi: 10.1038/s41598-023-42404-7 (PMC10555988; doi:10.1038/s41598-023-42404-7)

Supplementary Fig. 1

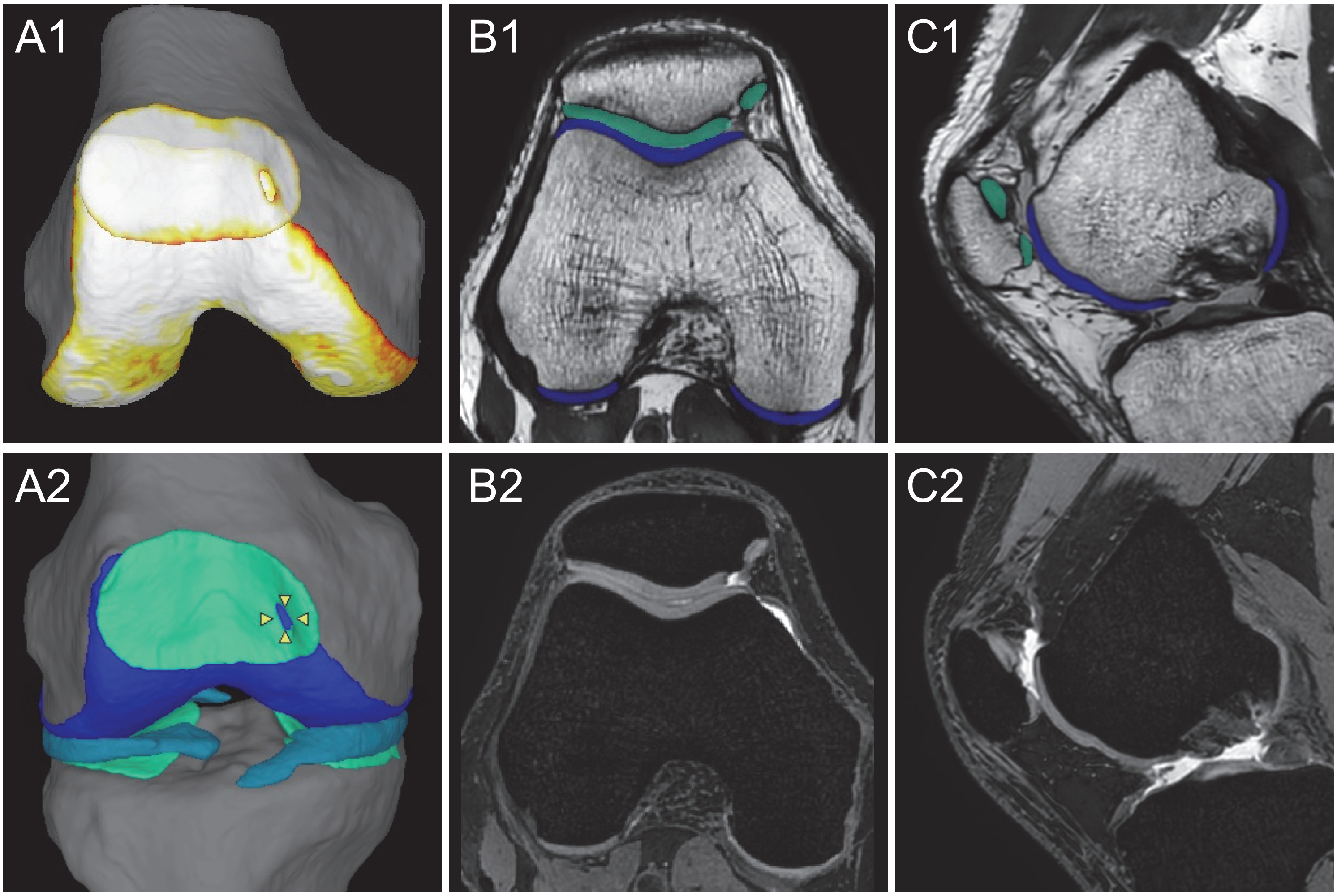

Supplementary Fig. 2-1 (continued)

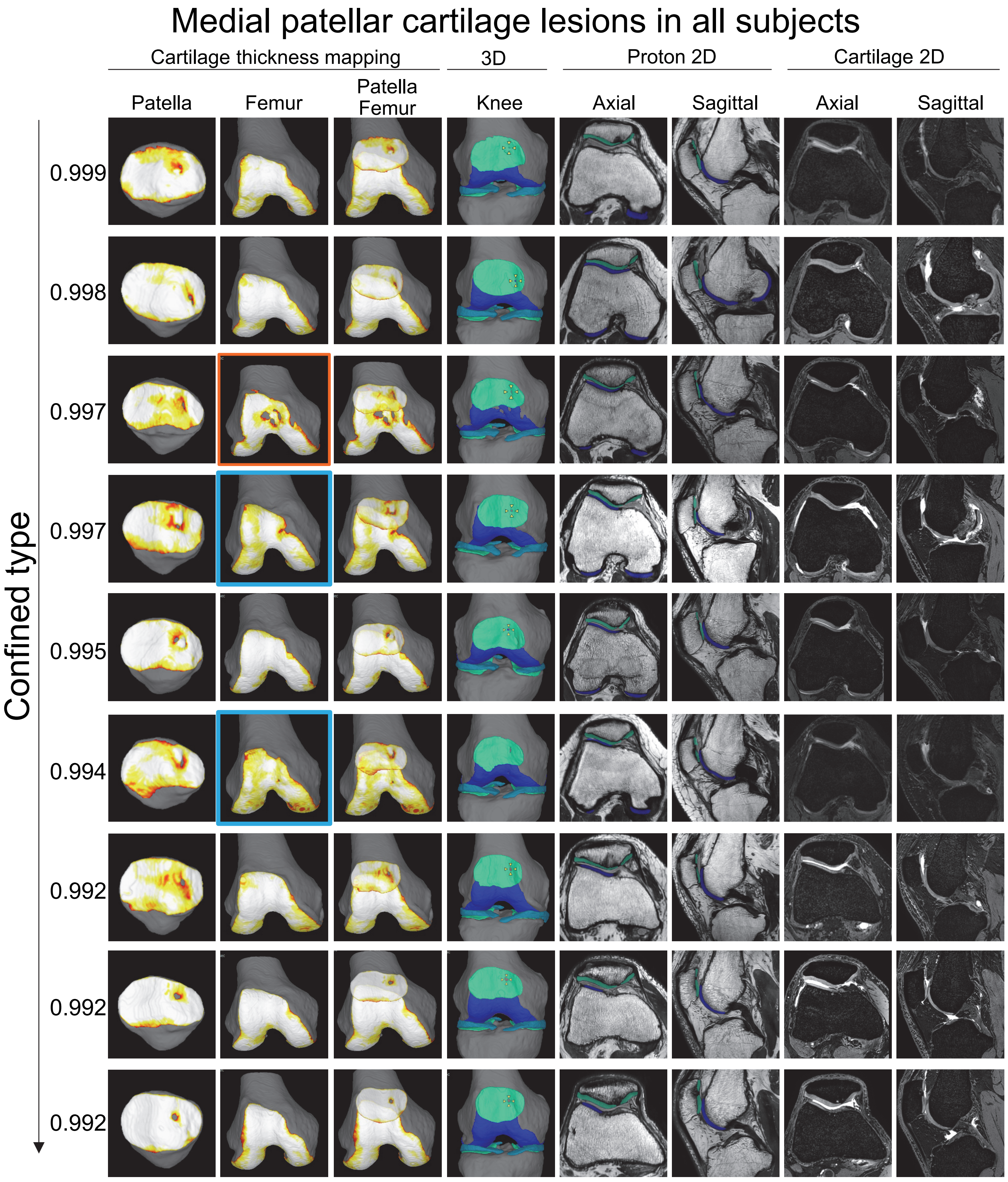

Supplementary Fig. 2-2 (continued)

Medial patellar cartilage lesions in all subjects

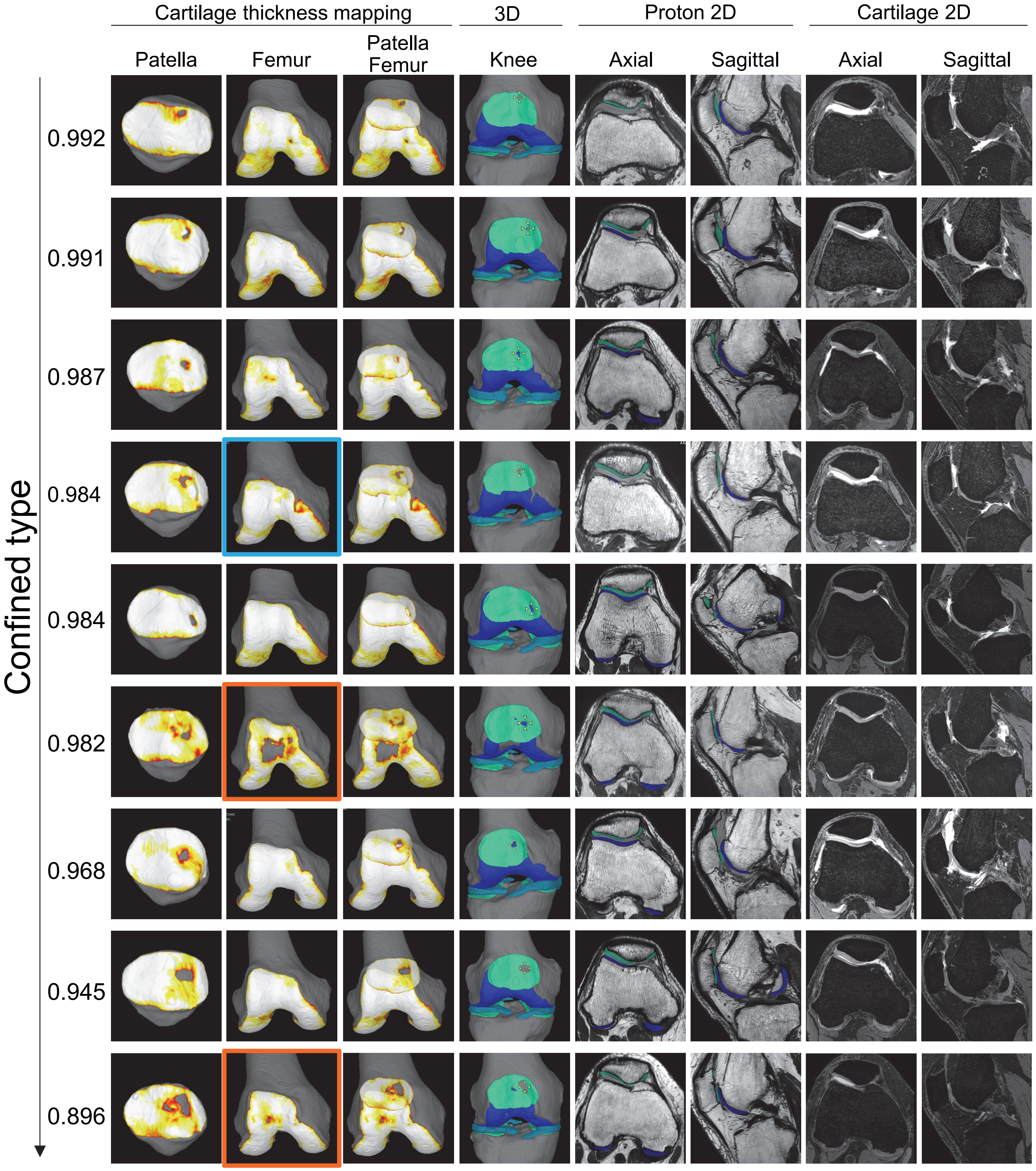

Supplementary Fig. 2-3

Medial patellar cartilage lesions in all subjects

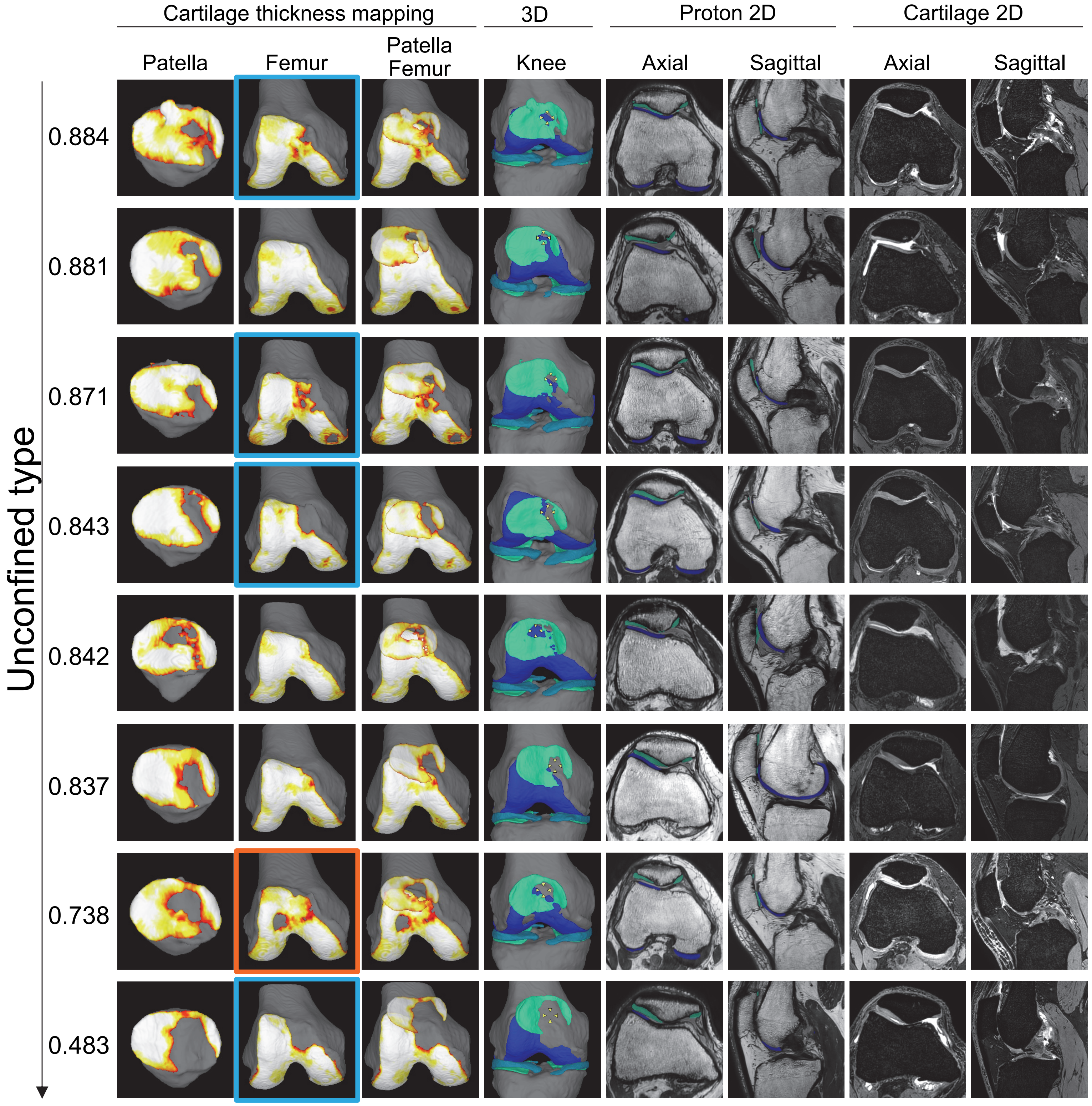

Supplementary Fig. 3

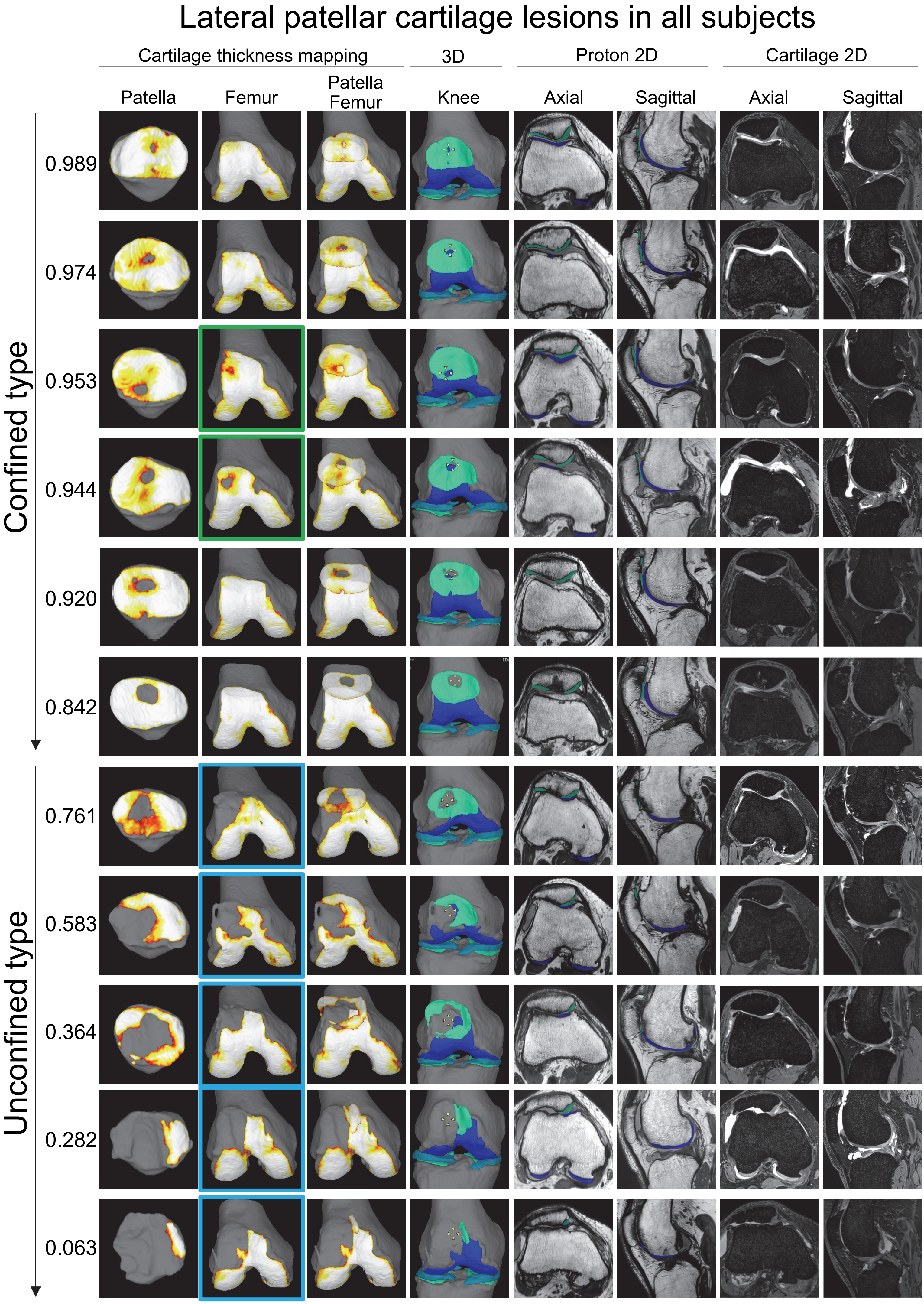

Supplement: Supplementary file 1 — Supplementary Figures. [file 41598_2023_42404_MOESM1_ESM.pdf]
