# Supplementary material for: Morphological analysis of three-dimensional MR images of patellofemoral joints in asymptomatic subjects
Source: Sci Rep. 2023 Oct 5;13:16750. doi: 10.1038/s41598-023-42404-7 (PMC10555988; doi:10.1038/s41598-023-42404-7)
Supplement: Supplementary file 2 — Supplementary Tables. [file 41598_2023_42404_MOESM2_ESM.docx]

**Supplementary Table 1**.

**Age and gender composition of subjects in the Kanagawa Knee Study.**

|  | Total (n=561) | |
| --- | --- | --- |
|  | Female  n=277 (100％) | Male  n=284 (100％) |
| Age |  |  |
| 30–39 years | 56 (20%) | 52 (18%) |
| 40–49 years | 61 (22%) | 62 (22%) |
| 50–59 years | 53 (19%) | 50 (18%) |
| 60–69 years | 55 (20%) | 57 (20%) |
| 70–79 years | 52 (19%) | 63 (22%) |

**Supplementary Table 2.**

**Number and incidence of patellar cartilage lesions by age and gender.**

|  | Total 37 (/561=7%) | |
| --- | --- | --- |
|  | Female  22 (/277=8%) | Male  15 (/284=5%) |
| Age |  |  |
| 30–39 years | 0 (/56=0%) | 0 (/52=0%) |
| 40–49 years | 0 (/61=0%) | 0 (/62=0%) |
| 50–59 years | 2 (/53=4%) | 2 (/50=4%) |
| 60–69 years | 8 (/55=15%) | 4 (/57=7%) |
| 70–79 years | 12 (/52=23%) | 9 (/63=14%) |
