# Supplementary material for: Morphological analysis of three-dimensional MR images of patellofemoral joints in asymptomatic subjects
Source: Sci Rep. 2023 Oct 5;13:16750. doi: 10.1038/s41598-023-42404-7 (PMC10555988; doi:10.1038/s41598-023-42404-7)
Supplement: Supplementary file 3 — Supplementary Legends. [file 41598_2023_42404_MOESM3_ESM.docx]

Supplementary Figure 1. Comparison between 3D and 2D MR images of patellar cartilage defects. (A) 3D MR images. Cartilage thickness mapping of the PF joint (1) and a 3D image of the knee joint (2). The patella cartilage is shown in green, femoral cartilage in blue, menisci in light blue, and tibial cartilage in green. The center of the cartilage defect in the patella is indicated by four arrowheads. (B) 2D axial images of the center of the cartilage defect. A proton density–weighted (PDW) image showing the patella cartilage in green and femoral cartilage in blue (1); a fat-suppressed spoiled gradient echo sequence (SPGR) image (2) is demonstrated. (C) 2D sagittal images showing the center of the cartilage defect. A PDW image (1) and SPGR image (2) are demonstrated.

Supplementary Figure 2. 3D and 2D MR images of the PF joints with cartilage defects in the medial region of the patella in all subjects. The center of the cartilage defect in the patella is indicated by four arrowheads in 3D. The center is depicted in each 2D MR image. The value on the left side indicates the cartilage area ratio, and each image is placed in order from the highest to lowest cartilage area ratio. Images framed in blue show femoral cartilage defects along the medial edge, and those framed in orange show the femoral cartilage defects at the center of the femoral trochlear cartilage.

Supplementary Figure 3. 3D and 2D MR images of the PF joints with cartilage defects in the lateral region of the patella in all subjects. The center of the cartilage defect in the patella is indicated with four arrowheads in 3D. The center is depicted in each 2D MR image. The value on the left side indicates the cartilage area ratio, and each image is placed in order from the highest to lowest cartilage area ratio. Images framed in green show that the femoral cartilage defect does not reach the edge of the trochlea, and those in blue show femoral cartilage defects along the lateral edge.
